# *VSX2* mutations in autosomal recessive microphthalmia

**Published:** 2011-09-28

**Authors:** Linda M. Reis, Ayesha Khan, Ariana Kariminejad, Farhad Ebadi, Rebecca C. Tyler, Elena V. Semina

**Affiliations:** 1 Department of Pediatrics and Children’s Research Institute at the Medical College of Wisconsin and Children’s Hospital of Wisconsin, Milwaukee, WI; 2 Al-Shifa Trust Eye Hospital, Rawalpindi, Pakistan; 3 Kariminejad-Najmabadi Pathology and Genetics Center, Tehran, Iran; 4 Diana Genetic Counseling Center, Kohkilooyeh Boyer Ahmad, Iran; 5 Department of Cell Biology, Neurobiology and Anatomy, Medical College of Wisconsin, Milwaukee, WI

## Abstract

**Purpose:**

To further explore the spectrum of mutations in the Visual System Homeobox 2 (*VSX2*/*CHX10*) gene previously found to be associated with autosomal recessive microphthalmia.

**Methods:**

We screened 95 probands with syndromic or isolated developmental ocular conditions (including 55 with anophthalmia/microphthalmia) for mutations in *VSX2*.

**Results:**

Homozygous mutations in *VSX2* were identified in two out of five consanguineous families with isolated microphthalmia. A novel missense mutation, c.668G>C (p.G223A), was identified in a large Pakistani family with multiple sibships affected with bilateral microphthalmia. This p.G223A mutation affects the conserved CVC motif that was shown to be important for DNA binding and repression activities of VSX2. The second mutation, c.249delG (p.Leu84SerfsX57), was identified in an Iranian family with microphthalmia; this mutation has been previously reported and is predicted to generate a severely truncated mutant protein completely lacking the VSX2 homeodomain, CVC domain and COOH-terminal regions.

**Conclusions:**

Mutations in *VSX2* represent an important cause of autosomal recessive microphthalmia in consanguineous pedigrees. Identification of a second missense mutation in the CVC motif emphasizes the importance of this region for normal VSX2 function.

## Introduction

*VSX2 *(Visual System homeobox 2, formerly known as *CHX10*) is a homeodomain containing transcription factor expressed in the developing retina in human [1], mouse [2], and zebrafish embryos [3,4]; *VSX2/Vsx2* deficiency results in microphthalmia with various associated ocular anomalies in all three species [1-3], suggesting that the function of this gene is evolutionarily conserved. The VSX2 homeoprotein is believed to mostly act as a repressor and, in some contexts, a weak activator, and utilizes the homeodomain and CVC domain in its interaction with DNA [5].

Mutations in *VSX2* are associated with autosomal recessive anophthalmia/microphthalmia (A/M), with or without iris coloboma and other ocular anomalies; eleven families have been described with eight different *VSX2* mutations [1,6-9]. In most cases, the ocular anomalies are isolated, but occasional extraocular features have been reported, including learning difficulties and hormone deficiency [9]. All affected individuals have homozygous mutations; to date, mutations have only been identified in consanguineous kindreds, primarily of West and South Asian background. Two previous studies failed to identify any *VSX2* mutations in 150 probands with A/M from Scotland [10] and 50 from Mexico [11].

A/M is a heterogeneous condition with numerous known causative genes. The most common causes of A/M are associated with autosomal-dominant inheritance and include SRY-Box 2 (*SOX2*) [12], Orthodenticle Homeobox 2 (*OTX2*) [13], and Bone Morphogenetic Protein 4 (*BMP4*)  [14] mutations. Several recessive alleles have also been reported. Homozygous and/or compound heterozygous mutations have been identified: in Forkhead Box E3 (*FOXE3*) in several families affected with nonsyndromic microphthalmia, often accompanied by aphakia and anterior segment anomalies [15-19]; in Retina and Anterior Neural Fold Homeobox Gene (*RAX*) in two probands with nonsyndromic anophthalmia [20,21]; and in Stimulated by Retinoic Acid 6 (*STRA6*) in syndromic A/M patients [22-24].

To further characterize the spectrum of *VSX2* mutations in A/M and other eye disease, we undertook screening of this gene in a large cohort of patients with various ocular conditions.

## Methods

This human study was approved by the Institutional Review Board of the Children’s Hospital of Milwaukee, WI with informed consent obtained by local physicians for every subject.

Genomic DNA was isolated from whole blood or buccal samples using standard procedures. The entire coding region and exon-intron junctions of *VSX2* (reference sequence NM_182894.2) were screened by direct DNA sequencing of PCR products in cases and controls, as previously described [17] with five sets of primers (Table 1). Briefly, PCR products were sequenced using 3730XL DNA Analyzer (Applied Biosystems, Foster City, CA) and results were analyzed manually and using Mutation Surveyor software (SoftGenetics, State Collge, PA). All initially identified changes were confirmed by additional independent PCR and sequencing experiments.

**Table 1:** Primer sequences and conditions for amplification of *VSX2* exons.

**Set/exon**	**Forward primer sequence**	**Reverse primer sequence**	**PCR product**	**Annealing temp/ special conditions**
Set 1/exon 1	TCCAGAGCATTAGACACCGG	TGGCAGGAACTTTTCCGCCT	603 bp	55 °C (5% DMSO; 20% Betaine)
Set 2/exon 2	GTTCAAAACCTCCGGATTCG	TCCGTTGTCGGCGAAAATAG	392 bp	55 °C
Set 3/exon 3	TCTTGTCTGAGACAGGCTCT	TCATGGGCATCTGGAACCCT	268 bp	55 °C (5% DMSO; 20% Betaine)
Set 4/exon 4	CACCATGGAGTAGGCGAGCT	ATTTCTCTCCTGCTAGGCTG	432 bp	55 °C
Set 5/exon 5	CAGTTCAAGATGGCTTTCCC	ATGTCTCAGCATGGTCCAGA	574 bp	55 °C

We screened 95 patients with ocular disorders, including 55 with A/M, 17 with anterior segment dysgenesis, 5 with coloboma, and 18 with other ocular conditions. There were 6 probands with autosomal recessive microphthalmia: all were consanguineous kindreds and five demonstrated multiple individuals with isolated microphthalmia in a clear recessive pattern. Race/ethnicity for the patients with anophthalmia/microphthalmia included Asian (7), African American (2), Caucasian (34), Hispanic (8), and other (4) and for the patients with other diagnoses included Asian (1), Caucasian (32), Hispanic (3), and other (4). Race-specific Caucasian and Asian control panels, as described previously [17], were screened for the identified mutations.

## Results

Homozygous mutations in *VSX2* were identified in two probands from consanguineous kindreds.

**Figure 1:**
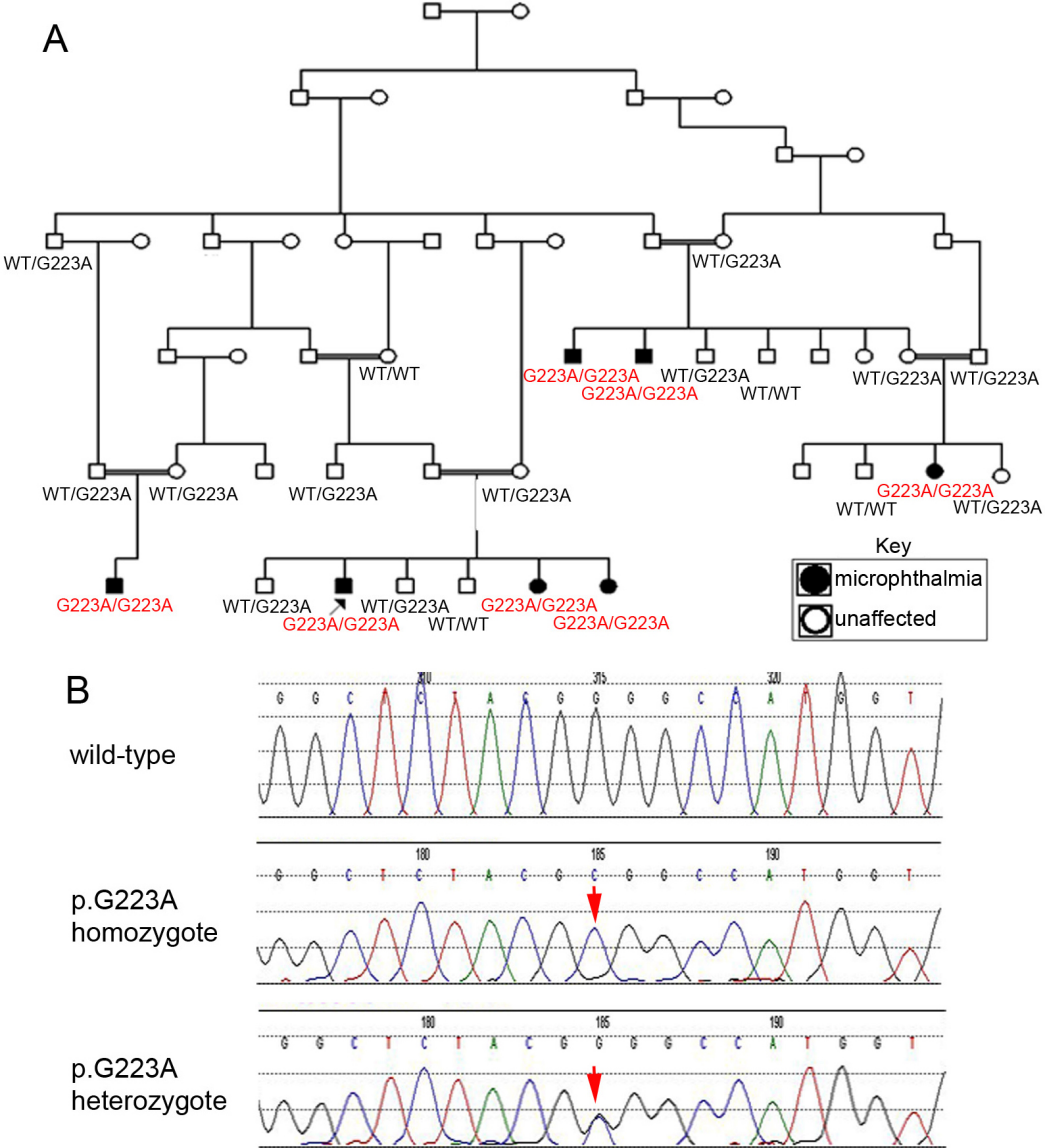
Pedigree and *VSX2* sequencing results for Patient 1 and family members. **A**: Patient 1 is indicated with a black arrow. *VSX2* genotype is indicated for each family member tested; genotypes of affected individuals are shown in red. WT, wild type. **B**: Mutation Surveyor view of forward *VSX2* sequencing data are shown; the position of the mutation is indicated with an arrow.

Patient 1 is an 11-year-old Pakistani male with isolated bilateral microphthalmia. He was found to have a homozygous c.668G>C (p.G223A) mutation, not previously reported. There is an extensive family history of consanguinity and microphthalmia; the mutation cosegregates with the disease phenotype with all affected individuals homozygous for the mutation and all tested unaffected individuals either heterozygous carriers or wild type (Figure 1).

**Figure 2:**
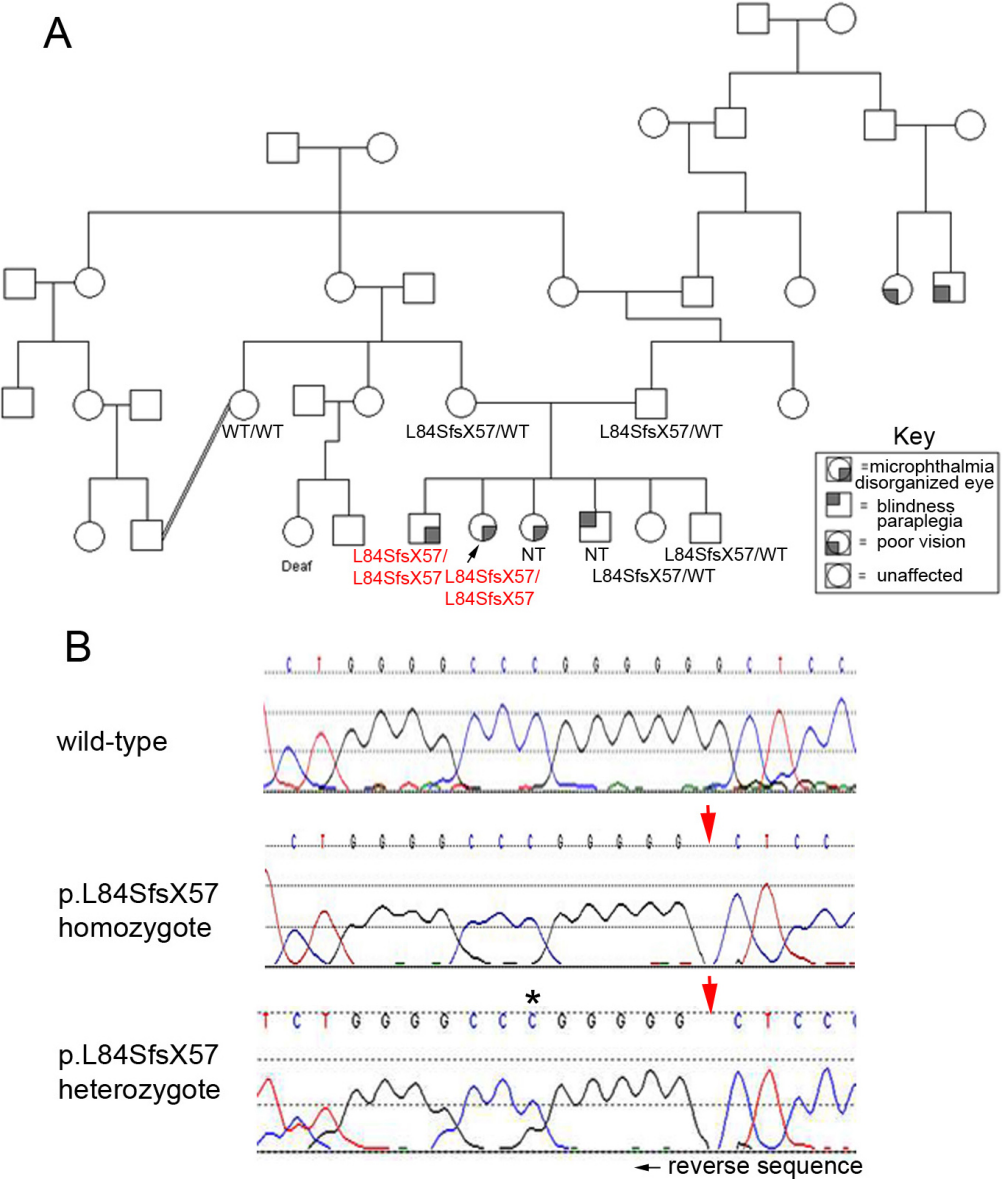
Pedigree and *VSX2* sequencing results for Patient 2 and family members. **A**: Patient 2 is indicated with a black arrow. *VSX2* genotype is indicated for each family member tested; genotypes of affected individuals are shown in red. WT, wild type; NT, not tested. **B**: Mutation Surveyor view of reverse *VSX2* sequencing data are shown; the position of the mutation is indicated with an arrow; the first position displaying the “phase shift” in the electropherogram trace which is characteristic of a heterozygous deletion is indicated with an asterisk.

Patient 2 is a 26-year-old Iranian female with bilateral microphthalmia, ‘disorganized eye,’ and blindness. She was found to have a homozygous c.249delG mutation (p.Leu84SerfsX57), previously reported [9].The parents are first cousins and there is a history of a similar phenotype in two siblings; the mutation cosegregates with the disease phenotype (Figure 2). An affected brother is homozygous for the mutation while the two unaffected siblings and the unaffected parents are heterozygous carriers and an unaffected maternal aunt is wild type. The other two siblings were not available for testing.

**Figure 3:**
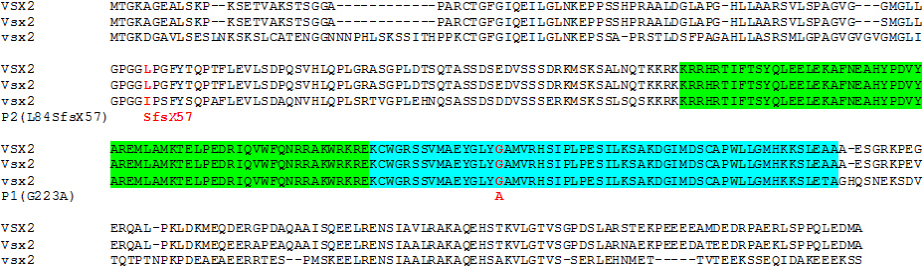
Alignment of protein sequences of human, mouse and zebrafish VSX2/Vsx2/vsx2. The homeodomain sequence is highlighted in green and the CVC motif in blue. The positions of the mutations identified in Patients 1 (P1) and 2 (P2) are marked in red.

Neither mutation was observed in control samples including 96 Asians and 94 Caucasian individuals. The first mutation is predicted to change a highly conserved amino acid inside the CVC-motif while the second mutation is predicted to result in a severely truncated mutant protein lacking the homeodomain, CVC-motif, COOH-terminal region, and a portion of the NH_2_-terminal arm (Figure 3).

## Discussion

These data confirm the role of *VSX2* in autosomal recessive isolated microphthalmia. Similar to previous reports, mutations were identified in consanguineous kindreds from Pakistan and Iran and no causative mutations were seen in probands with A/M from non-consanguineous kindreds. *VSX2* mutations were identified in 33% (2 out of 6) of consanguineous families with isolated microphthalmia.

The novel missense mutation seen in Patient 1, c.668G>C (p.Gly223Ala), is located within the conserved CVC motif, similar to the previously reported p.Arg227Trp mutation [6,9]. The absence of this mutation in controls and its perfect cosegregation with disease phenotype provides strong evidence that this change disrupts VSX2 function. The CVC motif is shared between the members of human VSX family, VSX1 and VSX2, as well as their numerous orthologs in other species. The glycine at position 16 of the CVC motif altered by this mutation is conserved in all known VSX proteins including the *C. elegans* protein ceh-10 [13]. The VSX2 homeodomain and CVC motif were demonstrated to be sufficient for DNA binding and repression; a deletion of the CVC motif resulted in a mild alteration of DNA binding but severely affected its repression ability [5].

This is the second report of the c.249delG mutation, previously reported in two sisters from Iran with microphthalmia, coloboma, and no perception of light [9]. Electroretinography (ERG) was performed on both parents in the previous report and demonstrated inner retinal dysfunction in both, suggesting a possible dominant effect for this mutation [9]. Unfortunately, we were unable to obtain ERG data for the heterozygous relatives of Patient 2 and thus cannot determine whether any mild retinal dystrophy is present in this family.

The *VSX2* mutations/phenotypes reported in this paper are consistent with the previously described *VSX2* spectrum. The absence of mutations in syndromic A/M cases is also in agreement with previous studies and further supports an eye-specific role for this gene in humans. This is only the second report of a missense mutation predicted to affect the *VSX2* CVC motif and resulting in a microphthalmia phenotype. The identification of this mutation emphasizes the importance of the CVC motif for normal VSX2 function and provides opportunities for further functional dissection.
